# On a Syndrome Characterised by Abnormally Low Blood Pressure, Bradycardia and Acrocyanosis

**Published:** 1909-08-28

**Authors:** 


### ON A SYNDROME CHARACTERISED BY ABNORMALLY LOW BLOOD PRESSURE, BRADYCARDIA AND ACROCYANOSIS.

There is a clinical condition with which many physicians must be familiar, but which has apparently escaped detailed notice until it was described recently by Dr. H. Vincent in Paris under the above title. It is of particular interest, perhaps, in that the patients seem to derive great benefit from treatment by thyroid extract; so much so, indeed, that Dr. Vincent would have us regard the condition as one of the many different types of hypothyroidism.

The patients generally present an appearance of intellectual apathy, with or without definite stigmata of degeneracy, and yet their general health seems good. Quite a number of them have either had acute rheumatism themselves, or else come of rheumatic stock. They do not develop the "syndrome" suddenly, but exhibit it more or less from childhood up. The patients are generally young, and usually female; quite a number are country girls.

Their extremities, particularly their hands, are apt to be cold, and discoloured a purplish-blue. There is often a remarkable smallness of the pulse wave, obvious to palpation as well as to the sphygmograph. Arterial tension, measured by some such instrument as that of Potain, Vaquez, or Riva-Rocci, is notably less than normal. Besides this, there is a remarkable fall in the pulse-rate when the patient lies down. Almost normal in frequency in the erect posture, it falls to 58, 52, 48, 42, and even 40 beats a minute in the horizontal, to rise again when the patient stands once more. It is, as Dr. Vincent terms it, a "*clino-static bradycardia*"; he has noticed a precisely similar condition in certain cases of subacute or resistant rheumatism. Normally, as Graves pointed out, there is a difference of 8 or 9 beats with a change of posture from standing to lying, and *vice versa*. Here the difference reaches 15, 20, 25 beats, or even more, according as the patient is examined standing up or lying down. At the same time the blood-pressure, already below normal, falls some 20 to 30 millimetres under the same circumstances.

As evidence of hypo-thyroidism in these cases, it is stated that palpation of the neck nearly always shows the thyroid gland to be deficient in size; there may even be an entire absence of one lobe. Moreover, the administration of thyroid gland extract ameliorates the circulatory phenomena in a few days in some cases, or in two or three weeks in others, the acrocyanosis, the bradycardia, and the arterial hypo-tension getting better rather more slowly. The improvement lasts a considerable time, and if the symptoms recur when the treatment is stopped they may be relieved again almost at once on repeating the thyroid gland extract administrations.

Dr. Vincent thinks there may be some connection between these cases and those of Raynaud's disease, and he suggests that thyroid extract might also benefit some of the patients suffering from the latter malady.

### The Sugar Test of Water Purity.

It is sometimes useful to be able to obtain an idea of the purity or otherwise of a given water supply without incurring the expense of a full chemical and bacteriological analysis. Among the constituents of sewage are phosphates in comparative abundance. If a clear-glass bottle is nearly filled with the water to be tested, a lump or two of sugar added, and the whole corked tightly and placed in a sunny place for two or three days, the water should remain quite clear. If, however, it contains phosphates in excess, a milkiness will have developed in it, in which case the suspicion of contamination would be sufficiently confirmed to warrant a full analysis of the water supply before any more of it was used for drinking purposes.

